# Effect of Fructooligosaccharide Metabolism on Chicken Colonization by an Extra-Intestinal Pathogenic *Escherichia coli* Strain

**DOI:** 10.1371/journal.pone.0035475

**Published:** 2012-04-13

**Authors:** Gaëlle Porcheron, Nathalie Katy Chanteloup, Angélina Trotereau, Annie Brée, Catherine Schouler

**Affiliations:** 1 INRA, UMR1282 Infectiologie et Santé Publique, Nouzilly, France; 2 Université François Rabelais de Tours, UMR1282 Infectiologie et Santé Publique, Tours, France; University of Osnabrueck, Germany

## Abstract

Extra-intestinal pathogenic *Escherichia coli* (ExPEC) strains cause many diseases in humans and animals. While remaining asymptomatic, they can colonize the intestine for subsequent extra-intestinal infection and dissemination in the environment. We have previously identified the *fos* locus, a gene cluster within a pathogenicity island of the avian ExPEC strain BEN2908, involved in the metabolism of short-chain fructooligosaccharides (scFOS). It is assumed that these sugars are metabolized by the probiotic bacteria of the microbiota present in the intestine, leading to a decrease in the pathogenic bacterial population. However, we have previously shown that scFOS metabolism helps BEN2908 to colonize the intestine, its reservoir. As the *fos* locus is located on a pathogenicity island, one aim of this study was to investigate a possible role of this locus in the virulence of the strain for chicken. We thus analysed *fos* gene expression in extracts of target organs of avian colibacillosis and performed a virulence assay in chickens. Moreover, in order to understand the involvement of the *fos* locus in intestinal colonization, we monitored the expression of *fos* genes and their implication in the growth ability of the strain in intestinal extracts of chicken. We also performed intestinal colonization assays in axenic and Specific Pathogen-Free (SPF) chickens. We demonstrated that the *fos* locus is not involved in the virulence of BEN2908 for chickens and is strongly involved in axenic chicken cecal colonization both *in vitro* and *in vivo*. However, even if the presence of a microbiota does not inhibit the growth advantage of BEN2908 in ceca *in vitro*, overall, growth of the strain is not favoured in the ceca of SPF chickens. These findings indicate that scFOS metabolism by an ExPEC strain can contribute to its fitness in ceca but this benefit is fully dependent on the bacteria present in the microbiota.

## Introduction

Extra-intestinal pathogenic *E. coli* (ExPEC) strains are responsible for a wide range of diseases in humans and animals. These strains have been isolated either from urinary tract infections, neonatal meningitis, cases of septicaemia of various origins, pneumonia, deep surgical wound infections and mastitis, or from colibacillosis in poultry, a systemic infection that starts in the respiratory tract [1]–[5]. This respiratory disease is characterized by fibrinopurulent lesions of internal organs such as air sacculitis, perihepatitis and pericarditis and is often associated with septicaemia and mortality [6], [7]. These extra-intestinal diseases represent a serious economic, medical and veterinary burden [8].

ExPEC strains can asymptomatically colonize the intestinal tract of humans and animals as commensal bacteria. Consequently, the intestine serves as a reservoir for pathogenic strains. Intestinal colonization by ExPEC is thus a potential risk factor for a subsequent extra-intestinal infection in the same host or for dissemination of pathogens in the environment, thus leading to a potential zoonotic risk [9]–[14]. The establishment of ExPEC in the intestine appears to play an important role in their future establishment in the urinary or respiratory tract. For instance, strains involved in urinary tract infections gain access to the periurethral area from the anus and establish infection in an ascending manner [15]. Strains involved in neonatal meningitis could translocate from the intestinal lumen of the neonate to the blood stream, and poultry inhale pathogenic *E. coli* in dust derived from faeces [6], [7], [16].

ExPEC strains mainly belong to the phylogenetical lineage B2 [2], [17]. It has been shown that strains belonging to this phylogenetic group have a greater ability to persist in the intestinal tract of healthy or infected people [18]–[21]. It has been suggested that virulence factors of ExPEC such as adhesins, or iron acquisition factors could confer a higher capacity to colonize their reservoir [18], [19], [22]. For example, the K5 capsule and P fimbriae enhance intestinal colonization in gnotobiotic rats [23], [24]. Moreover, the *frz* operon of the avian ExPEC strain BEN2908 and the pathogenicity islands of the uropathogenic strain 536 are fitness elements involved in intestinal colonization [25], [26]. Maintenance of intestinal colonization thus requires many properties, one of the most important being metabolic competence, in addition to virulence factors. When two strains compete for a limited nutrient, the one that is able to use it more efficiently should outcompete the other [26], [27].

We have recently shown that the ability of the strain BEN2908 to metabolize short-chain fructooligosaccharides (scFOS) enhances colonization of the chicken intestine by bacteria during the first 8 days post-inoculation [28]. scFOS are natural linear polymers comprising two to four β-(2-1)-linked fructosyl units, usually attached to a terminal glucose residue [29]. Like many complex plant carbohydrates, these sugars are not hydrolyzed by digestive enzymes, and therefore they reach the distal parts of the intestine intact where they are assimilated by the intestinal microbiota, particularly probiotic bacteria [30], [31].

The genomic region responsible for scFOS metabolism in the BEN2908 strain, called the *fos* locus (GenBank accession no. AY857617), is found on the AGI-3 pathogenicity island [32]. This locus is composed of six genes organized as an operon encoding for a putative MFS (Major Facilitator Superfamily) sugar transporter (FosT), two glycoside hydrolases of family 32 (FosE_1_ and FosE_2_), two proteins of unknown function (FosX and FosY), a fructokinase (FosK), and of a divergently transcribed gene encoding for a putative transcriptional regulator of the LacI/GalR family (FosR). We previously defined a regulatory model of scFOS metabolism in BEN2908 [33]. In the absence of scFOS, FosR is able to bind to the promoter of the *fos* operon on two operator sequences, suppressing *fos* gene expression. Moreover, *fos* gene expression relies on catabolite repression, and the presence of glucose represses this expression. It has also been shown that *fos* gene expression depends on the presence of scFOS in the medium.

Due to the presence of the *fos* locus of the ExPEC strain BEN2908 on the AGI-3 pathogenicity island, one aim of this study was to investigate a possible role of these genes in the virulence of BEN2908 for chickens by analysing *fos* gene expression in extracts of target organs of avian colibacillosis and by performing a virulence assay in chickens. Moreover, in order to study the involvement of the *fos* locus in the colonization of the strain's reservoir, we analyzed *fos* gene expression and the implication of these genes in the growth ability of BEN2908 in different intestinal extracts. Finally, we performed intestinal colonization assays in axenic and SPF (Specific Pathogen-Free) chickens. We found that the *fos* locus is not involved in the virulence of BEN2908 for chickens and that, even if this locus is strongly involved in axenic chicken intestinal colonization in the ceca, it does not significantly contribute to cecal colonization of SPF chickens.

## Results

### The *fos* locus is not involved in virulence

**Figure 1:**
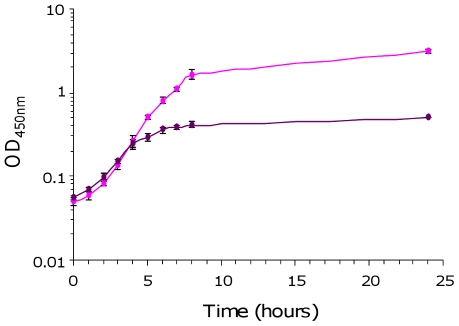
Growth of *E. coli* strain BEN2908 in the presence of scFOS at 37°C and 41.5°C. Growth curves (OD_450 nm_) of strain BEN2908 grown with shaking at 37°C (pink circle) or 41.5°C (purple circle) in M9 minimal medium supplemented with 0.2% scFOS. The average values and standard deviations result from three independent experiments.

As the *fos* locus is located on the AGI-3 pathogenicity island [32], we investigated a possible role of this locus in the virulence of the BEN2908 strain for chicken. Firstly, we examined *fos* gene expression in minimal media containing extracts of target organs of avian colibacillosis including the lung, liver, spleen and pericardial fluid. As the average body temperature of chicken is 41.5°C [34], we first checked the growth ability of the strain BEN2908 in M9 minimal medium containing scFOS at 41.5°C. Surprisingly, this strain was impaired in its ability to grow in this minimal medium at 41.5°C compaired to 37°C (Fig. 1). This impairment was also observed in M9 minimal medium containing glucose but not in a more complex medium such as LB-Miller medium (data not shown). As growth is altered in M9 minimal medium at 41.5°C, we thus performed the experiments at 37°C.

**Figure 2:**
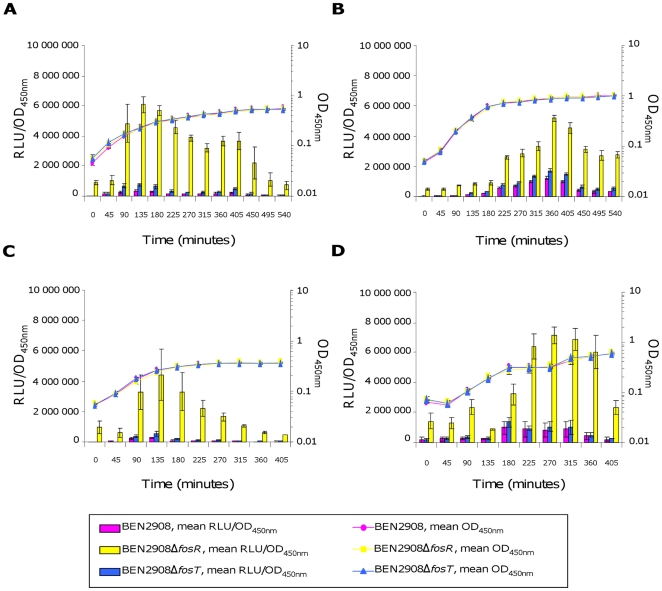
*fos* operon promoter activity in extracts of target organs of avian colibacillosis. Growth curves (OD_450 nm_) and relative luminescence intensities (RLU/OD_450 nm_) of strains BEN2908, BEN2908Δ*fosR* and BEN2908Δ*fosT* carrying pQF52 grown without shaking at 37°C in M9 minimal medium supplemented with (A) 10% of lung extract, (B) 4% of liver extract, (C) 4% of spleen extract, (D) 4% of pericardial fluid. The RLU average values and standard deviations result from three independent experiments.

**Figure 3:**
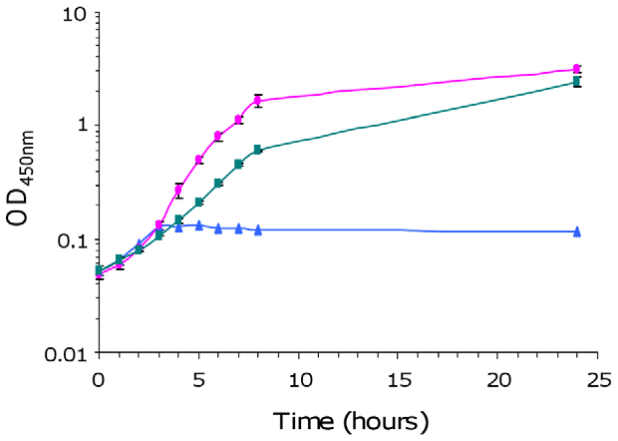
Growth of *E. coli* strains BEN2908, BEN2908Δ*fosT* and BEN2908Δ*fosT*/pGEM::*fos* in the presence of scFOS. Growth curves (OD_450 nm_) of strains BEN2908 (pink circle), BEN2908Δ*fosT* (blue triangle) and BEN2908Δ*fosT*/pGEM::*fos* (green square) grown with shaking at 37°C in M9 minimal medium supplemented with 0.2% scFOS. The average values and standard deviations result from three independent experiments.

**Figure 4:**
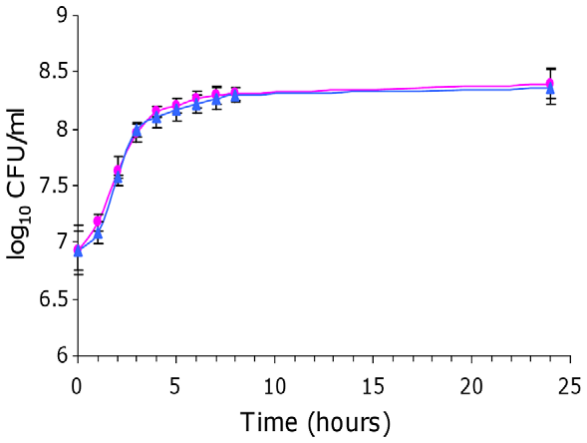
Competition between *E. coli* strains BEN2908 and BEN2908Δ*fosT* for growth in the presence of liver extract. Growth curves (log_10_ CFU/ml) of strains BEN2908 (pink circle) and BEN2908Δ*fosT* (blue triangle) grown without shaking at 37°C in M9 minimal medium supplemented with 4% of liver extract. The average values and standard deviations result from three independent experiments.

Expression of *fos* genes was monitored by measuring the *fos* promoter-mediated expression of luciferase from plasmid pQF52 during the growth of the BEN2908, BEN2908Δ*fosT* (unable to metabolize scFOS) and BEN2908Δ*fosR* (unable to repress *fos* gene expression) strains. As shown in Fig. 2, luciferase expression in BEN2908 and BEN2908Δ*fosT* was always lower than in BEN2908Δ*fosR* in all the media tested (5- to 31-fold higher in BEN2908Δ*fosR* than in BEN2908 in the lung, 3- to 14-fold higher in the liver, 14- to 36-fold higher in the spleen, and 3- to 14-fold higher in pericardial fluid; 5- to 17-fold higher in BEN2908Δ*fosR* than in BEN2908Δ*fosT* in the lung, 2- to 11-fold higher in the liver, 7- to 22-fold higher in the spleen, and 2- to 12-fold higher in pericardial fluid). This indicates that FosR repressed *fos* gene expression in these organ extracts. Nevertheless, *fos* genes were expressed slightly more in both BEN2908 and BEN2908Δ*fosT* in minimal media containing liver extract and pericardial fluid (a maximum of 1.22±0.11×10^6^ and 1.74±0.1×10^6^ RLU/OD_450_ in the liver, 1±0.42×10^6^ and 1.35±0.3×10^6^ RLU/OD_450_ in pericardial fluid, versus 3.34±0.4×10^5^ and 7.07±0.7×10^5^ RLU/OD_450_ in the lung and 2.7±0.3×10^5^ and 5.38±1.19×10^5^ RLU/OD_450_ in spleen extracts, respectively) (Fig. 2). This expression was not due to the presence in the media of sugars metabolized by the *fos* locus because it was not higher in BEN2908 than in BEN2908Δ*fosT* and there was no difference in the growth of these strains. We then verified that the *fos* locus, whose genes were slightly expressed in a minimal medium containing liver extract, did not give a growth advantage to strain BEN2908 in the presence of this colibacillosis target organ. Before, we checked that the impairment of the BEN2908Δ*fosT* strain in scFOS metabolism was only due to deletion of the *fosT* gene. We then introduced in this strain a pGEM-T derivative containing the whole *fos* locus [28]. As shown in Fig. 3, the recombinant BEN2908Δ*fosT* strain was able to grow with scFOS as the sole carbon source. However, this plasmid is unstable in M9 minimal medium containing organ extracts (70–80% of the recombinant bacteria maintain the plasmid during the growth, data not shown). As we performed co-cultures to determine the implication of the *fos* locus in the growth on such media, this instability did not allow us to realize complementation assays in the presence of organ extracts. To check the involvement of the *fos* locus in the presence of liver extract, BEN2908 and BEN2908Δ*fosT* were inoculated together in equal amounts in a minimal medium containing liver extract, and the proportion of each strain was monitored during their growth. As shown in Fig. 4, BEN2908 did not outcompete BEN2908Δ*fosT* in the presence of liver extract.

**Figure 5:**
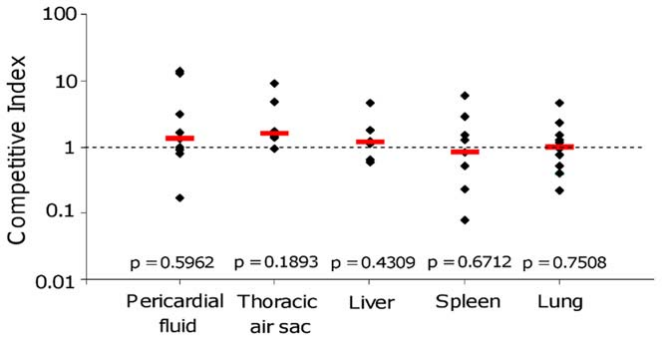
Competition between *E. coli* strains BEN2908 and BEN2908Δ*fosT* for colonization of chicken organs during avian colibacillosis. Twenty five-day-old SPF White Leghorn chickens were inoculated in the right air sac with strains BEN2908 and BEN2908Δ*fosT* together (each at 5×10^6^ CFU/chicken). Animals were euthanized 48 h post-inoculation by injection of Nesdonal and then necropsied. The proportion of each strain in organs was monitored and CI were calculated. Horizontal red bars indicate the median of CI and diamonds indicate individual CI. Statistical analyses were conducted using the Mann-Whitney U-test, measuring the difference between CI in organs and in the inoculum. The calculated *P* values are presented.

While these results strongly suggest that the *fos* locus is not involved in virulence, the expression of *fos* genes was not studied in blood and air sacs, which are other target organs/fluids of colibacillosis. We thus examined the impact of the *fos* locus on the virulence of the BEN2908 strain *in vivo*. To that end, equal amounts of BEN2908 and BEN2908Δ*fosT* were co-inoculated into the air-sac of SPF chickens (5×10^6^ CFU of each strain/chicken), and the proportion of each strain in organs was monitored. As shown in Fig. 5, there was no difference in the colonization of target organs of avian colibacillosis (lung, thoracic air sac, liver, spleen and pericardial fluid) by BEN2908 or BEN2908Δ*fosT*, as the competitive indexes did not differ significantly from 1. Overall, these results demonstrate that the *fos* locus is not involved in the virulence of BEN2908 for chicken.

### *fos* genes are expressed in chicken cecal content and not in intestinal mucus

**Figure 6:**
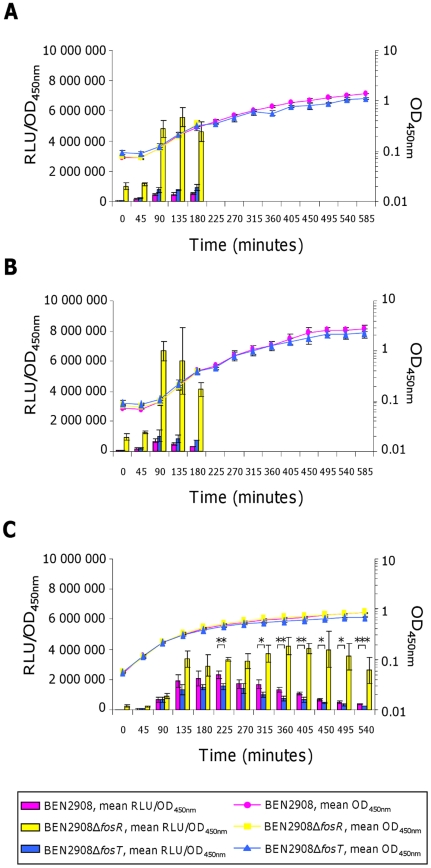
*fos* operon promoter activity in extracts of chicken intestine. Growth curves (OD_450 nm_) and relative luminescence intensities (RLU/OD_450 nm_) of strains BEN2908, BEN2908Δ*fosR* and BEN2908Δ*fosT* carrying pQF52 grown without shaking at 37°C in M9 minimal medium supplemented with (A) intestinal mucus from the ileum at 2 mg/ml of proteins, (B) intestinal mucus from the colon at 2 mg/ml of proteins, (C) 2% of cecal content. The RLU average values and standard deviations result from three independent experiments. Asterisks indicate significant differences in mean luciferase activity between BEN2908 and BEN2908Δ*fosT* determined by a Student's *t*-test. *** *P*<0.005; ** *P*<0.02; * *P*<0.05.

The involvement of the *fos* locus in chicken intestinal colonization has previously been demonstrated by monitoring the proportion of BEN2908 and BEN2908Δ*fosT* in faeces [28]. However, identifying viable bacteria in faeces does not enable the site(s) along the gastrointestinal tract in which the *fos* locus is important to be determined. We thus investigated whether *fos* genes were expressed in different parts of the distal intestine, i.e. in intestinal mucus collected from the ileum or colon and in cecal content of axenic chickens. As shown in Fig. 6A and B, luciferase expression in intestinal mucus from the ileum and colon was much lower in BEN2908 and BEN2908Δ*fosT* than in BEN2908Δ*fosR* (7.5- to 12-fold higher in BEN2908Δ*fosR* than in BEN2908 in the ileum, and 7- to 12.5-fold higher in the colon; 5- to 7-fold higher in BEN2908Δ*fosR* than in BEN2908Δ*fosT* in the ileum, and 5.5- to 7-fold higher in the colon). This indicates that no inducer was present in these media to lift the FosR repression and to allow *fos* gene expression. In cecal content, luciferase expression in BEN2908 and BEN2908Δ*fosT* was much higher (a maximum of 2.3±0.25×10^6^ and 1.53±0.2×10^6^ RLU/OD_450_ in cecal content, respectively, versus 5.32±0.49×10^5^ and 9.19±2.21×10^5^ RLU/OD_450_ in mucus from the ileum and 7.03±1.45×10^5^ and 1.05±0.4×10^6^ RLU/OD_450_ in mucus from the colon, respectively), although lower than expression in BEN2908Δ*fosR* (1.5- to 7-fold higher, and 1.5- to 11-fold higher in BEN2908Δ*fosR*, respectively) (Fig. 6C). This indicates that expression in BEN2908 and BEN2908Δ*fosT* was not fully activated. Moreover, growth of BEN2908Δ*fosT* was less than that of BEN2908 and BEN2908Δ*fosR* and luciferase expression was significantly higher in BEN2908 than in BEN2908Δ*fosT* at different times (Fig. 6C) [2.30±0.25×10^6^ and 1.53±0.2×10^6^ RLU/OD_450_ at 225 min (p = 0.014); 1.67±0.28×10^6^ and 9.92±1.94×10^5^ RLU/OD_450_ at 315 min (p = 0.027); 1.31±0.15×10^6^ and 7.51±1.46×10^5^ RLU/OD_450_ at 360 min (p = 0.01); 1.08±0.05×10^6^ and 6.71±1.63×10^5^ RLU/OD_450_ at 405 min (p = 0.014); 6.46±1.05×10^5^ and 4.64±0.32×10^5^ RLU/OD_450_ at 450 min (p = 0.045); 4.97±0.87×10^5^ and 3.32±0.44×10^5^ RLU/OD_450_ at 495 min (p = 0.042); 3.79±0.44×10^5^ and 2.43±0.23×10^5^ RLU/OD_450_ at 540 min (p = 0.009), respectively]. This suggests that inducers of *fos* gene expression are present in cecal content to support BEN2908 growth and that this strain is able to metabolize cecal nutrients via the *fos* locus.

**Figure 7:**
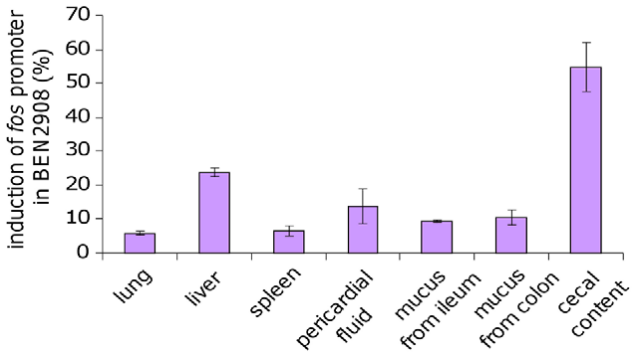
Activation of *fos* promoter in chicken extracts or physiological fluids. Percentage of *fos* promoter activation was obtained by dividing the maximum luciferase expression in BEN2908 by the maximum luciferase expression in BEN2908Δ*fosR*. The average values and standard deviations result from three independent experiments.

For a better comparison of activation of *fos* gene expression under all the conditions tested, we calculated the ratio of maximal luciferase activity in BEN2908 to the maximal luciferase activity in BEN2908Δ*fosR*. As shown in Fig. 7, maximal activation of the *fos* promoter was observed in cecal content (54.7±7.3%). The *fos* promoter was less activated in the presence of mucus from the ileum and colon (9.5±0.4% and 10.6±2.2%, respectively) than in the liver (23.7±1.4%), and was activated to the same extent in the presence of pericardial fluid (13.9±5.1%). It was activated least in the presence of lung and spleen extracts (5.8±0.5% and 6.5±1.5%, respectively).

### The *fos* locus is involved in growth on cecal content from axenic chicken *in vitro* and *in vivo*

**Figure 8:**
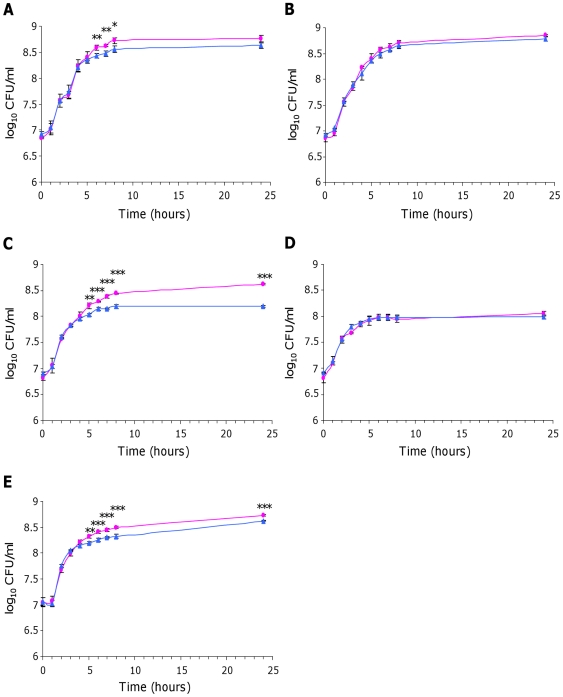
Competition between *E. coli* strains BEN2908 and BEN2908Δ*fosT* to grow in the presence of extracts of chicken intestine. Growth curves (log_10_ CFU/ml) of strains BEN2908 (pink circle) and BEN2908Δ*fosT* (blue triangle) grown without shaking at 37°C in M9 minimal medium supplemented with (A) intestinal mucus from the ileum at 2 mg/ml of proteins, (B) intestinal mucus from the colon at 2 mg/ml of proteins, (C) 2% of cecal content from axenic chicken, (D) 2% of cecal content from SPF chicken, (E) 4% of cecal content from axenic chicken supplemented with cecal bacteria from SPF chicken. The average values and standard deviations result from three independent experiments. Asterisks indicate significant differences in mean number of CFU between BEN2908 and BEN2908Δ*fosT* determined by a Student's *t*-test. *** *P*<0.005; ** *P*<0.02; * *P*<0.05.

As *fos* genes were expressed in cecal content, we then investigated whether the *fos* locus benefited BEN2908 growth in cecal content. We also investigated a possible role of this locus in the growth of BEN2908 on intestinal mucus from the ileum and colon. Indeed, although *fos* genes were not expressed in these media, BEN2908 grew better than BEN2908Δ*fosT* in these biological extracts when monitored independently during expression analyses (Fig. 6). To monitor the involvement of the *fos* locus in the growth of BEN2908 in the presence of samples from the distal intestine, equal amounts of BEN2908 and BEN2908Δ*fosT* were inoculated together into a minimal medium containing either intestinal mucus from the ileum or colon, or cecal content from axenic chickens. In the medium containing intestinal mucus from the ileum, there was no difference between BEN2908 and BEN2908Δ*fosT* during the exponential growth phase. From six hours of growth, strain BEN2908 had a significant advantage over BEN2908Δ*fosT* [8.59±0.04 and 8.43±0.05 log_10_ CFU/ml at 6 hours (p = 0.017); 8.63±0.02 and 8.48±0.06 log_10_ CFU/ml at 7 hours (p = 0.015); 8.73±0.06 and 8.56±0.06 log_10_ CFU/ml at 8 hours (p = 0.023), respectively]. However, at 24 hours of growth, this difference was no longer significant (Fig. 8A). In the medium containing intestinal mucus from the colon, there was no significant difference between BEN2908 and BEN2908Δ*fosT* during the entire growth curve (Fig. 8B). Finally, in the medium containing cecal content, there was a significant difference from five hours of growth with a distinct advantage of BEN2908 over BEN2908Δ*fosT* [8.21±0.06 and 8.03±0.02 log_10_ CFU/ml at 5 hours (p = 0.008); 8.28±0.02 and 8.14±0.03 log_10_ CFU/ml at 6 hours (p = 0.002); 8.38±0.03 and 8.14±0.02 log_10_ CFU/ml at 7 hours (p = 0.0003); 8.44±0.01 and 8.19±0.04 log_10_ CFU/ml at 8 hours (p = 0.0003); 8.62±0.02 and 8.2±0.01 log_10_ CFU/ml at 24 hours (p = 9.9×10^−6^), respectively] (Fig. 8C). These results indicate that the *fos* locus confers a slight growth advantage to BEN2908 in ileal mucus and a more marked advantage in cecal content from axenic chicken.

**Figure 9:**
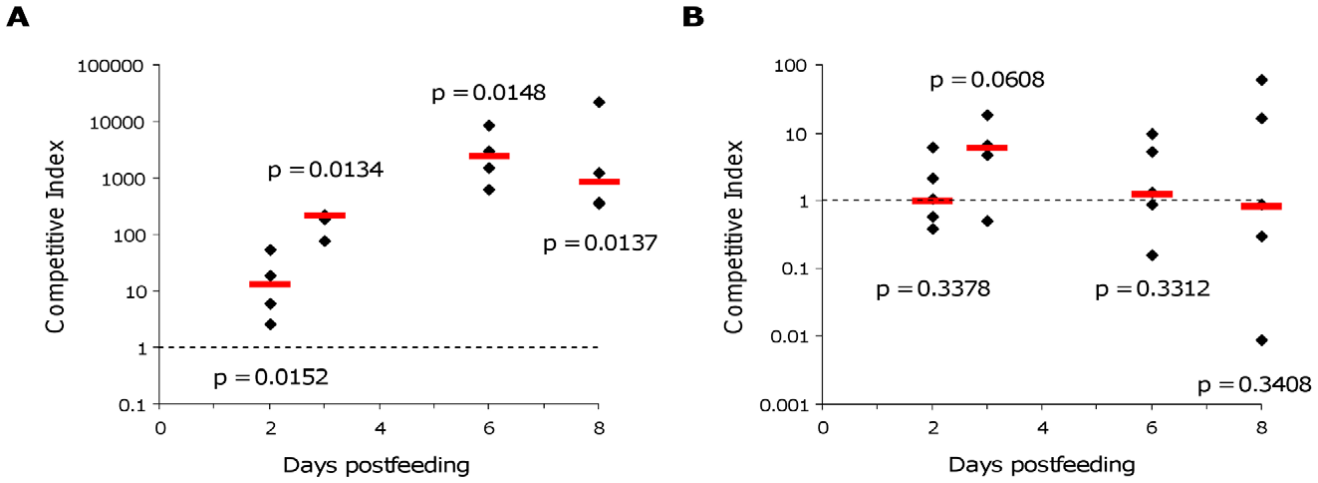
Chicken cecal colonization by *E. coli* strains BEN2908 and BEN2908Δ*fosT*. Eleven-day-old axenic (A) and SPF (B) White Leghorn chickens were fed with strains BEN2908 and BEN2908Δ*fosT* together (each at 5×10^7^ CFU/chicken). Animals were euthanized on days 2, 3, 6 and 8 post-inoculation by injection of Nesdonal and then necropsied. The proportion of each strain in animal cecal content was monitored over time and CI were calculated. Horizontal red bars indicate the median of CI and diamonds indicate individual CI. Statistical analyses were conducted using the Mann-Whitney U-test, measuring the difference between CI in cecal content and in the inoculum. The calculated *P* values are presented, with values below 0.05 considered significant.

To confirm this involvement in this intestinal compartment, we performed an *in vivo* experiment. BEN2908 and BEN2908Δ*fosT* were fed together in equal amounts to axenic chickens (5×10^7^ CFU of each strain/chicken), and the proportion of each was monitored in ceca. The results showed that the BEN2908 strain had a strong advantage in cecal colonization compared to the BEN2908Δ*fosT* strain up to eight days post-inoculation (median of CI of 12.73 at 2 days post-feeding; 215 at 3 days; 2382.5 at 6 days and 844.5 at 8 days) (Fig. 9A). This clearly indicates that the *fos* locus is involved in the colonization of axenic chicken ceca.

### The *fos* locus is involved in growth on cecal content in the presence of a microbiota *in vitro* but not *in vivo*

All the previous experiments were conducted with biological extracts from axenic chicken and do not represent the real conditions encountered by the bacteria *in vivo*. Indeed, sugars present in these extracts have not undergone any catabolism or degradation by bacteria. The sugar contents in the chicken intestine with a normal microbiota could be different. We thus analyzed the involvement of the *fos* locus in the growth of BEN2908 in cecal content from SPF chicken. When BEN2908 and BEN2908Δ*fosT* were inoculated together in a minimal medium containing previously sterilized cecal content from SPF chicken, there was no competition between the two strains, indicating that the sugars metabolized via the *fos* locus had previously been metabolized by the cecal microbiota (Fig. 8D). This also suggests that competition observed in cecal content from axenic chicken is a consequence of sugar metabolism mediated by the *fos* locus.

In order to avoid the use of sugars metabolized by the *fos* locus before sampling of cecal content but while looking at whether the presence of cecal microbiota could inhibit the benefit conferred by the *fos* locus, equal amounts of BEN2908 and BEN2908Δ*fosT* were inoculated together into a minimal medium containing cecal content from axenic chicken supplemented with cecal bacteria from SPF chicken. We first verified that these cecal bacteria were able to grow in a minimal medium containing cecal content from axenic chicken, although the experiments were not carried out in anaerobic conditions (8.97 log_10_ CFU/ml culturable on LB-Miller medium at 24 h) (data not shown). As shown in Fig. 8E, there was a significant difference after five hours of growth, with BEN2908 having a distinct advantage over BEN2908Δ*fosT* [8.32±0.03 and 8.2±0.02 log_10_ CFU/ml at 5 hours (p = 0.007); 8.41±0.02 and 8.26±0.04 log_10_ CFU/ml at 6 hours (p = 0.003); 8.45±0.03 and 8.3±0.02 log_10_ CFU/ml at 7 hours (p = 0.002); 8.50±0.03 and 8.32±0.04 log_10_ CFU/ml at 8 hours (p = 0.002); 8.73±0.02 and 8.63±0.004 log_10_ CFU/ml at 24 hours (p = 0.0005), respectively]. These results demonstrate that the BEN2908 strain is able to compete with a complex microbiota that also metabolizes the substrate of the *fos* locus.

We then checked if these results could be observed *in vivo*. To that end, BEN2908 and BEN2908Δ*fosT* were fed together in equal amounts to SPF chickens (5×10^7^ CFU of each strain/chicken), and the proportion of each was monitored in ceca. We also enumerated the total *E. coli* population in cecal content of each animal. This population varied from one animal to the other (from 3.41×10^5^ to 1.24×10^8^ CFU/g of cecal content) (Table 2). As shown in Fig. 9B, the BEN2908 strain had a slight advantage, but not significant, in cecal colonization compared to the BEN2908Δ*fosT* strain at 3 days post-feeding but no advantage was observed on other days (median of CI of 1 at 2 days post-feeding; 5.96 at 3 days; 1.24 at 6 days and 0.85 at 8 days) (Fig. 9B). These results indicate that, in the presence of a complex microbiota, the *fos* locus does not provide a benefit to the BEN2908 strain to colonize the intestine.

## Discussion

In this study, we demonstrated both *in vitro* and *in vivo* that the *E. coli* strain BEN2908 had a strong growth advantage in cecal content from axenic chicken over a strain that does not metabolize scFOS (Fig. 6C, 8C and 9A). In the presence of a microbiota in cecal content, we observed that, *in vitro*, the ability to metabolize scFOS gave significant competitive advantage to the BEN2908 strain (Fig. 8E). However, *in vivo* in SPF chickens, overall, no significant competitive advantage was observed but it is notable that competitive indexes considerably varied from one animal to another (Fig. 9B). As the cecum is an intestinal site where non-digestible carbohydrates such as cellulose, inulin and FOS are assimilated by the microbiota [35], [36], we can assume that the substrates giving a growth advantage to BEN2908 are scFOS. These results thus suggest that scFOS metabolism by the BEN2908 strain could contribute to its implantation in ceca. Nevertheless, one of the most important factors determining the nutrients found in the gastrointestinal tract is the presence of the intestinal microbiota. The number of bacteria found in the ceca of chicken is approximately 10^11^ CFU/g, including *Clostridiaceae*, *Sporomusa*, *Enterobacteriaceae*, *Fusobacterium*, *Bacteroides*, *Lactobacillus*, *Streptococcus*, *Ruminococcus* and *Bifidobacterium* [37]–[40]. The presence of these bacteria allows extensive bacterial fermentation, resulting in further nutrient absorption, detoxification of harmful substances and prevention of pathogen colonization [35], [38], [41]. Among these bacteria, some strains of *Lactobacillus*, *Bifidobacterium*, *Bacteroides* and *Fusobacterium prausnitzii* are able to metabolize nutrients such as FOS [37], [42]–[47]. These strains can therefore compete with the BEN2908 strain to metabolize FOS nutrients *in vivo*. We have previously shown that the *fos* locus of the BEN2908 strain was able to give an advantage to this strain to colonize the intestine of SPF chickens [28]. In this latter experiment, SPF chickens were obtained by inoculating axenic chickens with a complex bacterial inoculum consisting of a suspension of faeces from adult SPF hens that have been conserved during several years at −80°C. In this case, it is likely that there were no strict anaerobes in the faeces, therefore any anaerobes inoculated to chickens. Similarly, in the *in vitro* experiment performed in this study with the cecal content from axenic chickens supplemented with cecal bacteria from SPF chickens, it is possible that strict anaerobes did not survive during sampling of cecal content and/or did not survive in the medium because experiment was not achieve in anaerobic conditions. This suggests that a strict anaerobic bacterium is able to metabolize scFOS more efficiently than *E. coli* strain BEN2908.

During the intestinal colonization experiment in SPF chickens, we observed that the *E. coli* population varied from one animal to the other (Table 2). This suggests that, despite the fact that chickens had the same diet and same environmental conditions, the intestinal colonization by *E. coli* is highly variable. This fluctuation is also observed for the strains BEN2908 and BEN2908Δ*fosT*, the proportions of which in this total population were inconstant (from 0.07 to 61.43% for BEN2908 and from 0.01 to 25.62% for BEN2908Δ*fosT*) (Table 2). Finally, we can also observe that, in some animals, the BEN2908 strain was highly dominant in the total *E. coli* population (61.43%, 43.12%, 50.70% and 56.23% in chickens 75, 78, 83 and 85, respectively) whereas the BEN2908Δ*fosT* strain proportion was lower (a maximum of 25.62% in the chicken 94) (Table 2). This observation suggests that the *fos* locus, according to the bacteria present in the microbiota, and more particularly in the absence of a particular anaerobic bacterium able to metabolize scFOS most efficiently, is able to contribute to intestinal colonization. It can nevertheless be noted that, in few animals, and especially in chicken 90, the BEN2908Δ*fosT* strain is able to outcompete the BEN2908 strain (Table 2). As we have never observed this trend before, even in minimal media containing only one carbon source, we cannot currently explain this result in these animals.

It is noteworthy that the *E. coli* strain BEN2908 is fully able to colonize experimentally the chicken intestine, even in SPF animals. Conversely, commensal *E. coli* colonization cannot be studied experimentally in conventional animals due to colonization resistance, which results when all niches are filled by the microbiota. Streptomycin-treated animals are thus routinely used to study *E. coli* intestinal colonization [48]. The fact that BEN2908 is able to colonize SPF chicken intestine, without antibiotic treatment and on a long range, strongly suggests that ExPEC strains are better intestinal colonizers than commensal *E. coli* strains. This is consistent with previous observations demonstrating that strains belonging to phylogenetical lineage B2 have a greater ability to persist in the intestine [18]–[21].

In the present study, we investigated *fos* gene expression in different biological extracts and we showed that *fos* gene expression in cecal content was high in both the BEN2908 and BEN2908Δ*fosT* strain (Fig. 6C). This result is surprising since we previously showed that *fos* gene expression depends on the presence of scFOS [with β-(2-1) links] in the medium and that BEN2908Δ*fosT* is not able to metabolize these carbohydrates [28], [33]. Several hypotheses can therefore be considered. Different types of fructans exist: inulin and its derivatives, with one linear β(2-1)-linked fructosyl chain attached to the fructosyl residue of the sucrose starter; neo-series inulin with two linear β(2-1)-linked fructosyl chains, one attached to the fructosyl residue of the sucrose, the other to the glycosyl residue; levan with one linear β(2-6)-linked fructosyl chain attached to the fructosyl residue of the sucrose starter; and graminan with both β(2-1) and β(2-6) types of fructosyl linkages [49]. One interesting hypothesis is that another type of fructan induces *fos* gene expression. This inducer could enter via a transporter other than FosT, explaining *fos* gene expression in BEN2908Δ*fosT*. Another hypothesis to explain this result could be the action of an activator of *fos* gene expression. We previously demonstrated that *fos* gene expression also depends on catabolite repression and the binding of CRP-cAMP complex to the *fos* promoter region [33]. This binding enhances the ability of RNA polymerase to bind and initiate transcription of *fos* genes. cAMP synthesis is mediated by adenylate cyclase which is activated by phosphorylated EIIA^Glc^ (IIA component of the glucose-specific phosphoenolpyruvate:carbohydrate phosphotransferase system) [50]–[53]. The phosphorylation state of EIIA^Glc^ depends on the [phosphoenolpyruvate]/[pyruvate] ratio and is thus completely dependent on the substrates metabolized by the cell and differs according to the substrate [54]. In this study, *fos* gene expression in the BEN2908Δ*fosT* strain was up to 36% in cecal content and 33% in liver extract (compared to the expression in the BEN2908Δ*fosR* strain). Therefore, some substrates found in these media, entering via the FosT transporter in the BEN2908 and BEN2908Δ*fosR* strains, could lead to a lower [phosphoenolpyruvate]/[pyruvate] ratio and thus lower concentrations of phosphorylated EIIA^Glc^ and cAMP synthesis in these strains. This could also explain the high levels of expression in the BEN2908Δ*fosT* strain compared to the expression in the BEN2908Δ*fosR* strain. Another surprising result in this study is that *fos* gene expression was always lower in BEN2908 than in BEN2908Δ*fosR*, even in cecal content (Fig. 2 and 6). This indicates that in none of the biological extracts tested, corresponding to the conditions encountered by the bacteria *in vivo*, was there a sufficient inducer concentration to allow a complete lift of FosR repression, whereas previously we observed this situation *in vitro* [33].

When this study was initiated, the *fos* locus had not been identified in bacteria other than *E. coli* and its prevalence was low (only 10 of the 133 *E. coli* strains tested possessed this locus [28]). The subsequent release of newly discovered genome sequences in databases enabled us to identify a truncated locus similar to the *fos* locus in the genome of several pathogenic bacteria. Several *Klebsiella pneumoniae* strains and *Klebsiella variicola* strain At-22 possess a truncated locus including a transcriptional regulator sharing 77% identity with FosR, an MFS transporter sharing 85% identity with FosT, and a glycosyl-hydrolase sharing 73% identity with FosE_1_. This locus also comprises an intergenic region between the regulator and the transporter that is very similar to that of the *fos* locus (sharing 54% identity throughout the whole region), including the same regulatory elements of operator 1 and 2 sequences and a CRP-cAMP recognition sequence. Moreover, the *Enterobacteriaceae bacterium* 9_2_54FAA strain, isolated from inflamed biopsy tissue from a patient with Crohn's disease [*Enterobacteriaceae bacterium 9_2_54FAA* Sequencing Project, Broad Institute of Harvard and MIT (http://www.broadinstitute.org/)], possesses a truncated locus similar to the *fos* locus with a transcriptional regulator (73% identity with FosR), an MFS transporter (89% identity with FosT), a glycosyl-hydrolase (67% identity with FosE_1_), a protein of unknown function, and a fructokinase (66% identity with FosK). It is well known that the gastrointestinal tract of humans and animals is the reservoir of commensal and pathogenic *Enterobacteriaceae* strains such as *Klebsiella* [55]–[58]. This suggests that other pathogenic bacteria could assimilate scFOS and that this metabolism could be controlled by the same regulation mechanism as that of the *fo*s locus of BEN2908. Finally, we can thus postulate that, like the BEN2908 strain, the ability to metabolize scFOS could also enhance the ability of these pathogens to colonize their reservoir according to the bacteria present in the intestinal microbiota.

In sum, as the importance of the *fos* locus is dependent on the presence of the microbiota, and particularly on the presence of specific anaerobic bacteria, it could be of interest to study the involvement of this locus on intestinal colonization in different avian lineage and in chicken fed with different diets, thus with different intestinal microbiota, to determine if some conditions are more favorable to a pathogenic strain to colonize its reservoir then leading to greater dissemination of this strain in the local environment, dust for example, and hence colonization of the respiratory tract and pathogenesis.

## Materials and Methods

### Ethics statement

The housing, husbandry and slaughtering conditions complied with European Union guidelines for the care and use of laboratory animals. The experimental protocol for experimental colibacillosis was approved by the regional ethics committee [Comité d'Ethique en Expérimentation Animale (CEEA) Val de Loire] under number CL2007-44. The experimental protocol for intestinal colonization of chickens was approved by the regional ethics committee (CEEA Val de Loire) under number CL2007-43.

### Bacterial strains, plasmids and growth conditions

Bacterial strains and plasmids used in this study are listed in Table 1.

**Table 1:** Bacterial strains and plasmids used in this study.

Strain or plasmid	Relevant characteristics	Reference
*E. coli strains*		
BEN2908	Extraintestinal pathogenic strain; O2:K1:H5; Nal^r^ Fos^+^ Fim^+^ Iut^+^ IbeA^+^ AGI-3^+^, avian origin	[59], [60]
BEN2908Δ*fosT*	Isogenic deletion mutant of BEN2908; Nal^r^ Kan^r^ Fos^−^	[28]
BEN2908Δ*fosR*	Isogenic deletion mutant of BEN2908; Nal^r^ Fos^+^	[33]
*Plasmids*		
pQF52	*oripMB1 oripRO1600* Amp^r^; *luc* under the control of the *fos* promoter	[33]
pGEM::*fos*	pGEM-T easy vector containing the whole *fos* locus; Amp^r^	[28]

**Table 2:** Proportion of the BEN2908 and BEN2908Δ*fosT* strains in the total *E. coli* population in the cecal content of each SPF chicken.

Chicken	Days postfeeding	Total *E. coli* (CFU/g of cecal content)	Proportion of BEN2908 in the total *E. coli* population (%)	Proportion of BEN2908Δ*fosT* in the total *E. coli* population (%)	Competitive index
75	2	3.33×10^7^	61.43	9.11	5.92
76	2	1.00×10^8^	0.07	0.06	1.00
77	2	1.67×10^7^	2.57	6.07	0.37
78	2	1.68×10^7^	43.12	18.17	2.08
92	2	4.60×10^7^	5.95	9.41	0.56
79	3	2.25×10^7^	0.70	0.04	17.38
82	3	1.05×10^8^	5.59	0.78	6.28
83	3	1.46×10^7^	50.70	7.47	5.96
84	3	2.18×10^7^	2.76	5.07	0.48
91	3	4.36×10^7^	0.18	0.03	4.55
85	6	9.45×10^6^	56.23	9.90	4.99
87	6	6.85×10^6^	0.60	3.59	0.15
88	6	4.25×10^6^	0.45	0.47	0.84
89	6	9.09×10^6^	7.54	5.34	1.24
93	6	1.24×10^8^	0.11	0.01	9.10
80	8	1.85×10^7^	3.32	10.44	0.28
81	8	7.55×10^7^	1.85	0.03	56.05
86	8	3.05×10^7^	0.63	0.04	15.38
90	8	8.91×10^7^	0.10	10.71	0.01
94	8	3.41×10^5^	24.82	25.62	0.85

*E. coli* strain BEN2908, O2:K1:H5 is a nalidixic acid-resistant derivative of strain MT78 isolated from the trachea of a chicken with a respiratory infection [59], [60]. The *fosT* and *fosR* isogenic mutants of BEN2908 were also used in this study [28], [33]. Strains were routinely grown in LB-Miller medium at 37°C with agitation [61]. When necessary, antibiotics were used at the following concentrations: nalidixic acid 30 µg/ml, ampicillin 100 µg/ml, or kanamycin 50 µg/ml. For growth monitoring experiments, overnight LB-Miller cultures of strains were washed twice and resuspended in the same volume of M9 minimal medium [61]. The strains were then inoculated to an optical density (OD) at 450 nm of 0.05 and cultured at 37°C or 41.5°C with agitation in 20 ml of M9 medium supplemented with 0.2% scFOS (Profeed P95; Beghin Meiji, France). scFOS powder is a mixture containing small quantities of glucose, fructose and sucrose (5%), and larger amounts of kestose (37%), nystose (63%) and fructofuranosyl nystose (10%).

For expression analyses, overnight LB-Miller cultures of strains carrying the pQF52 plasmid [33] were washed as described previously. The strains were then inoculated to an OD at 450 nm of 0.05 and cultured at 37°C without agitation in 20 ml of M9 medium supplemented with either 2% of cecal content, 4% of liver or spleen extract, or 10% of lung extract, in 2.5 ml of M9 medium supplemented with 4% of pericardial fluid, or in 1 ml of M9 medium supplemented with intestinal mucus adjusted at a final protein concentration of 2 mg/ml as described by Edelman *et al.* [62]. In these experiments, biological extracts were collected from axenic chickens. For media containing pericardial fluid and intestinal mucus, bacterial growth was monitored by plating serial dilutions onto LB-agar plates supplemented with ampicillin at 100 µg/ml at different times.

For co-cultures of BEN2908 and BEN2908Δ*fosT*, overnight LB-Miller cultures were washed as described previously. The strains were then inoculated in equivalent numbers (each corresponding to an OD at 450 nm of 0.025) and cultured at 37°C without agitation in 10 ml of M9 medium supplemented with either 2% of cecal content from axenic or SPF chicken, 4% of cecal content from axenic chicken supplemented with cecal microbiota of SPF chicken, or 4% of liver extract, or in 0.3 ml of M9 medium supplemented with intestinal mucus adjusted to a final protein concentration of 2 mg/ml. At different times, serial dilutions were plated onto LB-agar plates supplemented with nalidixic acid at 30 µg/ml (selection of BEN2908 and BEN2908Δ*fosT* strains) or with kanamycin at 50 µg/ml (selection of the BEN2908Δ*fosT* strain) for bacterial quantification. The number of CFU of BEN2908 was calculated by subtracting the number of kanamycin-resistant bacteria from the number of nalidixic acid-resistant bacteria.

In all the experiments, the media supplemented with the biological extracts were vigorously vortexed, centrifuged for 5 min at 4,000× g, and supernatants were sterilized by filtration.

For the experiment with cecal bacteria, 100 µl of M9 minimal medium containing cecal microbiota were inoculated in 10 ml of M9 minimal medium supplemented with cecal content from axenic chickens (representing 5×10^5^ CFU/ml, culturable on LB-agar medium).

### Collection of chicken organs, pericardial fluid, cecal content, intestinal mucus and cecal microbiota

Liver, lung, spleen, pericardial fluid, cecal content and intestinal mucus were collected from 25-day-old axenic PA12 White Leghorn chickens from the Institut National de la Recherche Agronomique experimental platform for infectious diseases. Axenic PA12 White Leghorn chickens were obtained using the method described by Le Bars [63]. Cecal content was also collected from 25-day-old SPF PA12 White Leghorn chickens. None antibiotic resistant *E. coli* strain was present in the intestine of SPF chickens. Animals were euthanized by Nesdonal (Rhône-Mérieux, Lyon, France) injection in the occipital sinus. Organs were collected and homogenized in sterile saline. Pericardial fluid was collected with a Pasteur pipette and ceca were collected and drilled to recover cecal content. Aliquots containing extracts of organ, pericardial fluid or cecal content from axenic chicken were stored at −20°C. Mucus was isolated from the ileum and from the colon of chicken as described previously [64], [65]. The animals were fasted 20 h before isolating intestinal mucus. Briefly, colon and ileum were collected and gently rinsed with PBS to remove intestinal content. Mucus was isolated from the intestinal walls by gentle scraping with the back of a scalpel, diluted 1∶3 with 25 mM HEPES (pH 7.2 to 7.5; Invitrogen) and vigorously vortexed. Epithelial cells and large cell components were removed by centrifugation at 11,000× g for 10 min at 4°C. The supernatant was then centrifuged at 26,000× g for 15 min at 4°C, and aliquots of the supernatant containing the crude mucus were stored at −20°C. The protein concentration of the mucus preparation was determined using a Bradford protein assay (Biorad).

Cecal content from SPF chicken was preserved in glycerol at −80°C to conserve cecal bacteria. To collect cecal microbiota, this cecal content was washed to remove cecal matter while maintaining cecal bacteria. To that end, one volume of cecal content was first diluted 1∶10 in M9 minimal medium and centrifuged 1 min at 200× g. Supernatant was recovered, centrifuged for 1 min at 300× g, then 2 min at 300× g. Finally, the supernatant was centrifuged for 2 min at 16,000× g and the pellet was resuspended with the same volume of M9 minimal medium.

### Luciferase measurements

Promoter activities of the *fos* operon in different media were determined by firefly luciferase expression along the growth curve as described previously [33]. Briefly, samples of 100 µl were taken every 45 min, and light emission (relative light unit, RLU) was recorded with a luminometer (Lumat LB 9507, Berthold). A luciferase Assay System kit (Promega) was used with some modifications. Samples were incubated with 300 µl of freshly lysed buffer [1× CCLR (Cell Culture Lysis Reagent, Promega), 1.25 mg/ml lysozyme (Sigma), 2.5 mg/ml BSA (Sigma)] for 10 min with agitation at room temperature. Solutions were quick-frozen in liquid nitrogen and then immediately incubated at 37°C. After thawing, samples were incubated for 10 min with agitation at room temperature. Finally, RLU was measured by incubating 25 µl of cell lysate with 50 µl of Luciferase Assay Reagent.

To compare levels of luciferase expression, a correlation curve between OD at 450 nm and the number of bacterial CFU was plotted for each strain. Numbers of CFU obtained in media containing pericardial fluid and intestinal mucus were then converted into OD values to normalize results.

### Experimental colibacillosis

An *in vivo* virulence assay was conducted as described previously with some modifications [32]. SPF chickens were obtained from the Institut National de la Recherche Agronomique experimental platform for infectious diseases. Fifteen 25-day-old SPF PA12 White Leghorn chickens were inoculated in the right thoracic air sac with a 0.1 ml suspension containing a mixture of equal numbers of BEN2908 and BEN2908Δ*fosT* (each approximately at 5×10^6^ CFU) in LB-Miller medium. The inoculum was prepared from an overnight culture of each strain grown in 20 ml LB-Miller medium at 37°C without agitation. Animals were euthanized 48 h post-inoculation by injection with Nesdonal (Rhône-Mérieux, Lyon, France) in the occipital sinus and necropsied. A swab of the left thoracic air sac was taken, and samples of the left lung, liver, spleen and pericardial fluid were collected. After homogenization in sterile saline, serial dilutions were plated onto Drigalski agar plates (Biorad) supplemented with nalidixic acid at 30 µg/ml (selection of BEN2908 and BEN2908Δ*fosT* strains) or with kanamycin at 50 µg/ml (selection of the BEN2908Δ*fosT* strain) for bacterial quantification. The number of CFU of BEN2908 was calculated by subtracting the number of kanamycin-resistant bacteria from the number of nalidixic-resistant bacteria. Competition indices (CI) were calculated following Freter et al.'s method using BEN2908 as the reference strain [CI = (number of BEN2908 CFU/number of BEN2908Δ*fosT* CFU)/(number of BEN2908 CFU/number of BEN2908Δ*fosT* CFU in the initial inoculum)] [66]. By definition, a CI of >1 indicates out-competition of the mutant strain (BEN2908Δ*fosT*) by the wild-type reference strain (BEN2908). A CI equal to 1 indicates no difference in colonization of organs, and a CI of <1 indicates out-competition of the wild-type reference strain (BEN2908) by the mutant (BEN2908Δ*fosT*).

### Intestinal colonization of chickens

An intestinal colonization assay was conducted as described previously with some modifications [28]. Briefly, 16 axenic and 20 SPF 11-day-old PA12 White Leghorn chickens from the Institut National de la Recherche Agronomique experimental platform for infectious diseases were fed with a 0.5 ml suspension containing a mixture of equal numbers of BEN2908 and BEN2908Δ*fosT* (each approximately at 5×10^7^ CFU) in LB-Miller medium. None antibiotic resistant *E. coli* strain was present in the intestine of SPF chickens. The inoculum was prepared from an overnight culture of each strain grown in 20 ml LB-Miller medium at 37°C without agitation. Animals were euthanized on days 2, 3, 6 and 8 post-inoculation by injection with Nesdonal in the occipital sinus and necropsied. Ceca were collected and cecal contents were weighed and then homogenized in sterile saline (9 ml/g of cecal content). Viable *E. coli* cells were counted by plating 10-fold dilutions in sterile saline on Drigalski agar plates (Biorad) supplemented with nalidixic acid at 30 µg/ml (selection of BEN2908 and BEN2908Δ*fosT* strains) or with kanamycin at 50 µg/ml (selection of the BEN2908Δ*fosT* strain). The number of CFU of BEN2908 was calculated by subtracting the number of kanamycin-resistant bacteria from the number of nalidixic-resistant bacteria. The numbers of cecal CFU were calculated per gram of cecal content. Competition indices (CI) were calculated as described before for experimental colibacillosis.

In the intestinal colonization experiment of SPF chicken, total *E. coli* population of cecal contents was counted by plating 10-fold dilutions of cecal contents in sterile saline on Drigalski agar plates (Biorad). In this experiment, we also verified that the kanamycin-resistant *E. coli* population was also nalidixic acid-resistant and unable to metabolize scFOS. To that end, kanamycin-resistant colonies were picked on LB-Miller agar plates containing nalidixic acid at 30 µg/ml. All the colonies tested were nalidixic acid-resistant. Moreover, for each animal, kanamycin-resistant colonies were pooled, resuspended in 10 ml of LB-Miller medium containing kanamycin at 50 µg/ml and incubated overnight at 37°C with agitation. Overnight LB-Miller cultures were washed twice in M9 minimal medium and resuspended in the same volume of M9 minimal medium. Five milliliters of M9 minimal medium supplemented with 0.2% scFOS were then inoculated with 50 µl of the washed culture and incubated at 37°C with agitation. No growth was observed for any of the pools tested.

### Statistical analysis

Statistical analyses of CI were performed using the Mann-Whitney U test. Exact *P* values were calculated with StatXact software (version 5.0; Cytel Inc., Cambridge, MA). Statistical analyses of luciferase expression and growth ability were performed using a Student's *t*-test.
